# Evaluation of Nailfold Capillaroscopic Findings in Pediatric Patients with Celiac Disease: A Cross-Sectional and Comparative Study

**DOI:** 10.3390/diagnostics15162102

**Published:** 2025-08-21

**Authors:** Gül Çirkin, Raziye Burcu Taskin

**Affiliations:** 1 Department of Pediatric Gastroenterology, Hepatology and Nutrition, Health Sciences University, Tepecik Training and Research Hospital, Izmir 35020, Turkey; gul_cirkin@hotmail.com; 2 Department of Pediatric Rheumatology, Health Sciences University, Tepecik Training and Research Hospital, Yenişehir Neighborhood, Gaziler Street Number: 468, Izmir 35020, Turkey

**Keywords:** celiac disease, pediatrics, nailfold videocapillaroscopy, gluten, dietary

## Abstract

**Background/Objectives:** Celiac disease (CD) is a chronic autoimmune enteropathy with increasing recognition of systemic involvement, including potential microvascular alterations. While nailfold videocapillaroscopy (NVC) is an established tool in rheumatology for assessing microcirculation, its application in pediatric CD remains unexplored. Our aim was to investigate capillaroscopic abnormalities in children with CD and assess their associations with clinical and laboratory parameters, including dietary adherence. **Methods:** This cross-sectional study included 76 pediatric CD patients and 76 age- and sex-matched healthy controls. All participants underwent standardized NVC evaluation, assessing capillary density, dilatation, morphology, and microhemorrhages. Clinical data, laboratory values, and dietary adherence (based on clinical symptoms and tissue transglutaminase-IgA levels) were recorded. **Results:** Compared to controls, CD patients exhibited significantly lower capillary density and increased frequencies of dilated capillaries, microhemorrhages, and abnormal morphologies (*p* < 0.001). A nonspecific NVC pattern predominated among CD patients. Capillary abnormalities were more pronounced in patients without tTG-IgA normalization (>10 U/mL) and with symptoms suggestive of gluten exposure. Additionally, the number of dilated capillaries positively correlated with age and disease duration. No significant differences were found based on ANA status. **Conclusions:** This is the first study to demonstrate NVC-detectable microvascular alterations in pediatric CD. Findings suggest subclinical microvascular involvement, which may be potentially modifiable through dietary adherence. NVC may serve as a non-invasive tool to detect early vascular changes and monitor systemic manifestations in pediatric CD. Longitudinal studies are warranted to clarify the reversibility and prognostic implications of these abnormalities.

## 1. Introduction

Celiac disease (CD) is an autoimmune condition, which can be triggered by gluten intake in genetically predisposed individuals, and results in chronic damage to the small intestinal mucosal barrier [1]. Beyond involvement in the gastrointestinal tract, it is now recognized that celiac disease has a broader impact on multiple organ systems, including an increased risk of cardiovascular manifestations [2]. Furthermore, it is frequently accompanied by other autoimmune diseases, such as connective tissue disorders, which themselves confer an elevated risk of vascular involvement [1,3]. However, the risk of vascular involvement in children with CD remains controversial.

Recent experimental data from a humanized mouse model suggest that CD induces systemic vascular alterations through activation of intestinal immunity [4]. Although direct human evidence is limited, these findings imply that CD may contribute to microvascular dysfunction and endothelial injury. Supporting this view, several studies have demonstrated that there is an increased risk of the development of microvascular complications in children with concomitant type 1 diabetes mellitus (T1DM) and CD, regardless of their glycemic control status [5]. In addition, recent pediatric evidence has indicated vascular alterations, such as increases in carotid intima-media thickness and arterial stiffness, which can occur in individuals with CD, even in the absence of elevated blood pressure [6]. Nonetheless, the current body of evidence on CD-associated vasculopathy remains limited, restricting the development of specific vascular screening protocols.

Nailfold videocapillaroscopy (NVC) is a rapid, non-invasive, cost-effective technique that allows direct visualization of the microcirculation. It has recently gained prominence as a valuable diagnostic and prognostic tool in the field of rheumatology, especially for monitoring patients with Raynaud’s phenomenon who are at risk of developing autoimmune disorders [7,8]. This technique is commonly applied in both the initial evaluation and longitudinal follow-up of juvenile systemic sclerosis (jSSc) and juvenile dermatomyositis (JDM). Beyond rheumatological diseases, its application has been extended to various non-rheumatological conditions associated with inflammation-mediated microvascular dysfunction, such as hypertension, diabetes mellitus, and Crohn’s disease [9,10,11]. Nonetheless, there is a notable gap in the literature concerning pediatric populations with celiac disease, with existing knowledge primarily derived from a single adult study [12].

In light of the possible microvascular alterations associated with celiac disease, we aimed to test the hypothesis that microvascular changes detected by NVC in children with celiac disease are more prevalent than in healthy controls and are associated with non-compliance with a gluten-free diet.

## 2. Materials and Methods

### 2.1. Study Design and Participants

From January 2024 to June 2025, a cross-sectional comparative study was conducted in the Pediatric Gastroenterology and Rheumatology Departments of Tepecik Training and Research Hospital to investigate whether microvascular involvement is present in CD and to assess its association with clinical features, particularly adherence to a gluten-free diet. Pediatric patients aged <18 years who were diagnosed with CD in the Pediatric Gastroenterology Department of Tepecik Training and Research Hospital and had no accompanying systemic illnesses were included. The diagnoses of the patients were made according to the guidelines of the European Society for Pediatric Gastroenterology, Hepatology and Nutrition (ESPGHAN). Accordingly, patients with serum tissue transglutaminase (tTG) immunoglobulin A (IgA) (tTG-IgA) levels exceeding 10 U/mL underwent upper gastrointestinal endoscopy. Duodenal biopsy samples were obtained and evaluated using the Marsh classification, and only those with Type 3 lesions were included in the study [13,14].

Inclusion criteria also required that the diagnosis be established at our center, disease onset occur before 18 years of age, tTG-IgA values be available at both diagnosis and at the most recent follow-up visit (when the capillaroscopy assessment was performed), and that patients had provided consent to participate in the study. To avoid potential confounding factors, patients with systemic autoimmune connective tissue diseases such as systemic lupus erythematosus (SLE), juvenile Sjögren’s syndrome (jSS), and jSSc that could influence nailfold capillaroscopic findings were excluded after a detailed rheumatological history and examination. Patients with coexisting T1DM, selective IgA deficiency, or incomplete records were also excluded from the study.

To compare the NVC findings, a healthy control group was formed with the same number of children who had no history of systemic disease or gastrointestinal symptoms and showed no evidence of growth retardation, selected from those attending the same hospital outpatient clinic for routine health examinations. However, for ethical reasons, invasive procedures or serological testing for celiac disease were not performed in these asymptomatic children.

### 2.2. Data Collection

Data on demographics, clinical features, baseline laboratory results, and histopathology from diagnosis to the last visit were systematically recorded using standardized forms, with all assessments performed by a pediatric gastroenterologist. Demographic variables included sex, current age, age at CD diagnosis, duration of follow-up, and family history. Clinical data encompassed gastrointestinal symptoms (diarrhea, constipation, abdominal pain, distension, bloating, vomiting, and weight loss) and extraintestinal symptoms (irritability, fatigue/lethargy, rash, and joint pain/inflammation). Anthropometric parameters included weight (kg), height (cm), and body mass index (BMI) standard deviation score (SDS). Among the baseline laboratory investigations assessed at diagnosis, erythrocyte sedimentation rate (ESR), C-reactive protein (CRP), prothrombin time (PT), international normalized ratio (INR), and antinuclear antibody (ANA) levels were recorded. All laboratory tests were performed in the hospital’s central laboratory, and tTG-IgA levels were measured using the enzyme-linked immunosorbent assay (ELISA) method.

The rheumatologist who performed the NVC was blinded to the participants’ group allocation. NVC was conducted without knowledge of whether the subject was a patient or control. Subsequently, the same rheumatologist, still blinded, obtained a rheumatologic history and performed a physical examination for connective tissue diseases in all participants.

At the last visit, when capillaroscopy was performed, all patients underwent clinical evaluation, physical examination (including anthropometric measurements), and tTG-IgA testing. Based on tTG-IgA levels and clinical findings from this assessment, patients were categorized into two groups: Group 1 included those without tTG-IgA normalization (>10 U/mL) and with symptoms suggestive of gluten exposure, while Group 2 included those with normalized tTG-IgA (≤10 U/mL) and no symptoms [15].

### 2.3. Nailfold Capillaroscopy Assessment

NVC was performed using a standardized protocol as previously described in the literature [16,17]. All evaluations were conducted under standardized environmental conditions. Before image acquisition, the study participants rested for at least 20 min in a quiet room with an ambient temperature of 20–24 °C. A single experienced rheumatologist, blinded to the clinical status of the subjects, performed all the examinations using a Dino-Lite Capillary Scope (MEDL4N Pro, AnMo Electronics Corporation, Hsinchu, Taiwan) with ×200 magnification and fiberoptic illumination. It was ensured that none of the children in either the patient group or the control group had undergone any dermatological or cosmetic procedures involving the nailfold area within 15 days before the nailfold capillaroscopy assessment.

NVC was applied to eight fingers of each participant, excluding the thumbs. Two adjacent images were obtained from each finger, yielding a total of sixteen images per subject. Image acquisition and analysis were conducted using DinoXcope software (version 2.6).

The capillaroscopic parameters listed below were evaluated according to internationally accepted definitions [16,17].

- Capillary density: The number of capillaries per linear millimeter; a density of <7/mm was considered abnormal.
- Capillary morphology:
  - Normal: Hairpin-shaped, crossing once or twice, or tortuous capillaries with a convex tip.
  - Abnormal: Branching or bushy morphology, features suggestive of neoangiogenesis, non-convex tips, or capillaries exhibiting three or more crossings.
- Capillary dilations: Capillaries with an apical loop diameter of 20–50 μm (normal: <20 μm).
- Giant capillaries: Homogeneously dilated capillaries with an apical diameter of ≥50 μm.
- Microhemorrhages: Presence or absence of punctate or flame-shaped hemorrhagic spots in the pericapillary area.

For quantitative analysis, the mean values of capillary density, number of dilated capillaries, tortuous forms, and capillary morphology, including abnormal shapes and normal features such as capillaries with crossings or tortuosity, were calculated by averaging values across all 16 images per participant. Giant capillaries and microhemorrhages were noted as either present or absent.

For qualitative analysis, each image was categorized as one of the following.

- Scleroderma pattern: The presence of giant capillaries and/or multiple architectural irregularities, typically accompanied by a significant decrease in capillary density. (≤3/mm).
- Non-scleroderma patterns:
  - Normal pattern: Homogeneous capillary size (<20 μm), normal morphology, and capillary density ≥7/mm.
  - Nonspecific pattern: Features including abnormal morphology, presence of dilations, or a density <7/mm without fulfilling criteria for the scleroderma pattern.

Classification was determined based on the image findings. If at least one image demonstrated a scleroderma pattern, the subject was assigned to that category. In the absence of scleroderma findings, the predominant non-scleroderma pattern (normal or nonspecific) was recorded. In cases where both non-scleroderma patterns were equally represented, the nonspecific pattern was prioritized.

### 2.4. Ethical Approval and Informed Consent

Approval for this study was granted by the Ethics Committee of İzmir Tepecik Training and Research Hospital (Decision No: 2025/01-16, Date: 5 February 2025). The parents or legal guardians of all the children provided written informed consent for participation in the study.

### 2.5. Statistical Analysis

Data obtained in the study were analyzed statistically using IBM SPSS Statistics for Windows, version 25.0 (IBM Corp., Armonk, NY, USA, 2017) Descriptive statistics were stated as the mean ± standard deviation (SD) or median and interquartile range (IQR: 25th–75th percentiles) values, depending on their distributional characteristics for continuous variables and as number (*n*) and percentage (%) for categorical variables. The conformity of continuous variables to a normal distribution was examined using the Kolmogorov–Smirnov test. In the comparisons of two independent groups of data, the Mann–Whitney U test was used when the data did not show a normal distribution or when sample sizes were small (n < 30). The Kruskal–Wallis H test was applied in the comparisons of three or more independent groups. When this test indicated significant differences, the Dunn test with Bonferroni correction was used as post hoc analysis to control for multiple comparisons. Relationships between categorical variables were examined using the Pearson Chi-Square test, Fisher’s exact test, or Yates’ correction, depending on data characteristics and expected frequencies. The relationships between capillaroscopic parameters and clinical variables in the celiac disease group were examined with Spearman’s rank correlation coefficient (Spearman’s rho), as these data did not exhibit a normal distribution. A value of *p* < 0.05 was accepted as the level of statistical significance.

## 3. Results

### 3.1. Demographic and Clinical Features of the Participants

The study enrolled 76 children diagnosed with CD [median age: 13 years (range: 4–18); 57.9% female] and 76 age- and gender-matched healthy control subjects [median age: 11 years (range: 5–17); 48.7% female]. There were no significant differences between the groups in terms of age or sex (*p* > 0.05). The BMI SDS values were significantly lower in the CD group compared to the control group (*p* = 0.005).

Group 1 patients, those without tTG-IgA normalization (>10 U/mL) and with symptoms suggestive of gluten exposure, accounted for 57.9% of the CD cohort, and ANA positivity was observed in 13.2% of patients (Table 1).

### 3.2. Nailfold Videocapillaroscopy Findings

#### 3.2.1. Capillaroscopic Differences Between CD Patients and Healthy Controls

Quantitative analysis of NVC demonstrated a significantly lower median capillary density in the CD group compared to the control group (*p* < 0.001). A markedly higher prevalence of dilated capillaries (*p* < 0.001) and microhemorrhages (*p* = 0.006), and an increase in capillary length (*p* = 0.026) were determined in the CD group.

In terms of capillary morphology, a significantly higher rate of both abnormal shapes (*p* < 0.001) and normal morphological variants, such as crossing (*p* < 0.001) and tortuous capillaries (*p* < 0.001), was observed in the CD group than in the control group.

**Figure 1:**
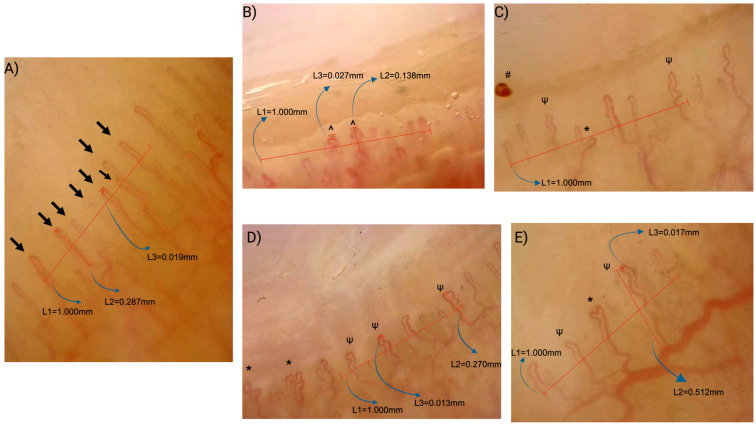
Examples of nailfold videocapillaroscopy findings in healthy controls (**A**) and patients with celiac disease (**B**–**E**). (**A**) Healthy control: Normal hairpin-shaped capillaries with normal density (8 capillaries/mm; ≥7/mm), without dilation, abnormal shapes, or microhemorrhages, representing a normal pattern. (**B**) Celiac disease: Two crossing capillaries (^) and one dilated capillary (>20 µm, <50 µm) as shown in L3 (27 µm), with reduced density (6/mm), representing a nonspecific pattern. (**C**) Celiac disease: Abnormal shaped capillary (*), tortuous capillaries (ψ) and microhemorrhage (#) with normal density (7/mm), representing a nonspecific pattern. (**D**) Celiac disease: Abnormal shaped capillaries (*) and tortuous capillaries (ψ) with normal density (7/mm), representing a nonspecific pattern. (**E**) Celiac disease: Abnormal-shaped capillary (*) and tortuous capillary (ψ) with reduced density (6/mm), representing a nonspecific pattern. Measurement references: L1—grid representing 1 mm nailfold in real life; L2—capillary length; L3—apical loop diameter.

Qualitative evaluation showed a significantly higher rate of nonspecific NVC patterns in the CD group (*p* < 0.001). The detailed capillaroscopic findings are presented in Table 2, with capillaroscopy image examples shown in Figure 1.

#### 3.2.2. Correlations and Subgroup Analyses

**Figure 2:**
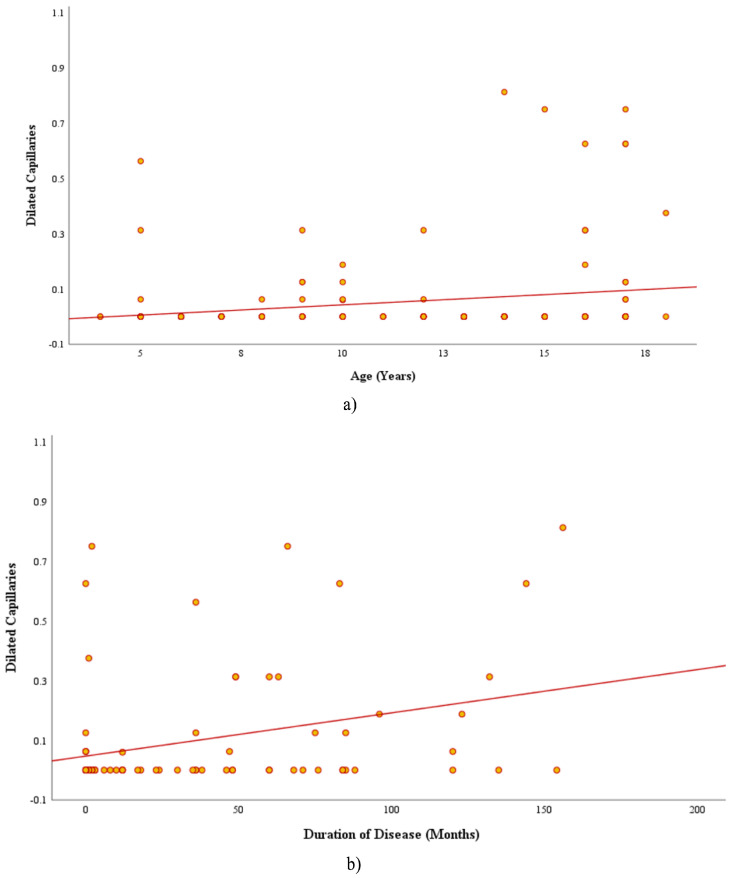
The correlation between the number of dilated capillaries and both (**a**) age (r = 0.228, *p* = 0.048) and (**b**) disease duration (r = 0.252, *p* = 0.028).

When the CD patients were analyzed according to disease duration (newly diagnosed ≤3 months vs. established diagnosis >3 months) or ANA positivity, there were observed to be no significant differences in the capillaroscopic findings (*p* > 0.05 for all). As a result of the correlation analyses, a statistically significant positive relationship was determined between the median number of dilated capillaries and both age (r = 0.228, *p* = 0.048) and disease duration (r = 0.252, *p* = 0.028) (Figure 2). There were not seen to be any other significant correlations between other capillaroscopic parameters and clinical or demographic variables, including BMI-SDS and tTG-IgA levels.

#### 3.2.3. The Effect of Adherence to a Gluten-Free Diet on Capillaroscopic Parameters

When the CD patients were grouped according to tTG-IgA levels and clinical symptoms, the median counts of dilated, tortuous, and crossing capillaries were seen to be significantly lower and capillary density was notably higher in the control group than in either of the CD subgroups.

The highest median number of capillaries with abnormal morphology was observed in patients from Group 1, significantly surpassing both the good-adherence group and the control group. Group 2 also displayed a higher frequency of abnormal capillary morphology compared to the control group. In terms of capillary density, dilated capillaries, crossing patterns, or tortuosity, there was seen to be no significant difference between Groups 1 and 2. These capillaroscopic findings are presented in detail in Table 3.

#### 3.2.4. Capillaroscopic Findings According to Antinuclear Antibody Status

Comparisons of the capillaroscopic parameters showed no significant differences between the ANA-negative (*n*:66) and ANA-positive (*n*:10) CD patients (*p* > 0.05 for all). Although the ANA-negative group exhibited a higher mean capillary length (284.9 ± 75.7 µm) compared to the ANA-positive group (249.9 ± 55.8 µm), the difference was not at a statistically significant level (*p* = 0.057). Other parameters, including capillary density, dilatation, abnormal shape, crossing, and tortuosity, were seen to be similar in both groups, and there was found to be no significant difference with respect to inflammatory markers such as ESR, CRP, PT, or INR (*p* > 0.05).

## 4. Discussion

Although NVC is commonly used to assess microvascular architecture in autoimmune conditions, its application in detecting comparable vascular alterations in children with CD remains largely unexplored in the literature. In this context, the current study can be considered to provide the first insights into NVC patterns observed in pediatric CD, addressing a gap that has not been previously explored. The observed reduction in capillary density, together with higher rates of dilated loops, microhemorrhages, and morphological abnormalities, suggests that possible microvascular alterations occur throughout the disease, even in the absence of clinically apparent vasculitis or systemic autoimmune involvement.

Specific NVC patterns are well characterized only in jSSc; however, nonspecific capillary abnormalities such as decreased density, dilated loops, and microhemorrhages have also been reported in pediatric SLE, JDM, and other connective tissue diseases [9,16,17,18]. Such microvascular alterations can emerge even when Raynaud’s phenomenon is not clinically evident, suggesting that overlapping immune-related mechanisms may underlie various autoimmune disorders [19]. Consistent with this perspective, the current study findings revealed that children with CD exhibited a markedly increased frequency of these nonspecific capillary changes. We also observed a positive correlation between the number of dilated capillaries and both patient age and disease duration, indicating that microvascular changes may progress over time. Considering that pediatric CD has not been previously evaluated in this context, the only available evidence in adults comes from a cross-sectional study comparing newly diagnosed active celiac disease, remission cases, and HCs [12]. That study reported capillary abnormalities in 60% of active cases—most often capillary density loss, avascular areas, dilated capillaries, and occasionally microhemorrhages or tortuosity—without giant capillaries, disorganization, extravasation, or abnormal shapes. In contrast, we observed abnormal capillary shapes in our patient group, possibly related to the inclusion of individuals with longer disease duration, as well as reduced capillary density, dilated, tortuous, and crossing capillaries in the healthy control group, consistent with reports that such findings may also occur in healthy children [20]. Taken together, our results provide a detailed quantitative evaluation of NVC parameters in a pediatric population and offer novel insights into the potential microvascular involvement in celiac disease during childhood.

Another key finding of this study was the significant difference in abnormal capillary morphology according to dietary adherence. In CD, the absence of a gold standard method for assessing adherence to a GFD (gluten-free diet) presents a significant challenge, as patients must maintain lifelong gluten-free nutrition [15]. Currently, the best available non-invasive adherence marker is tTG-IgA; however, normalization of tTG serology may take more than a year, particularly in individuals with high initial titers or severe mucosal injury, limiting its sensitivity as an early adherence indicator [21]. In our cohort, participants without tTG-IgA normalization (>10 U/mL) exhibited the highest frequency of capillary morphological abnormalities, whereas even those with normalized tTG-IgA (≤10 U/mL) had higher counts than healthy peers. These findings may reflect ongoing gluten exposure and greater microvascular disruption, warranting further investigation. Previous study has demonstrated elevated serum levels of endothelial adhesion molecules (VCAM-1, ICAM-1, and E-selectin) in pediatric CD, with partial normalization following GFD compliance [22]. Although both biochemical markers and NVC alterations may reflect shared underlying endothelial involvement in CD, the cross-sectional design of the present study precludes establishing a direct cause-and-effect relationship. Longitudinal studies are needed to assess whether sustained GFD adherence leads to NVC normalization and if follow-up NVC, combined with tTG-IgA levels, could serve as an additional tool for monitoring adherence in clinical practice.

Since the capillary alterations identified in our study have also been described in other autoimmune conditions, they are not unique to celiac disease. Their presence supports the hypothesis that the autoimmune nature of the disease may predispose affected children to microvascular abnormalities. CD triggers a wide range of immune-mediated reactions. One hypothesized pathway is that exposure to gliadin impairs the intestinal barrier function, allowing immunologically active peptide fragments to enter the bloodstream. This process activates tissue transglutaminase 2 and initiates a Th1-driven immune response, leading to the generation of autoantibodies and immune complexes [23], which may contribute to microvascular damage. Given that CD is more frequently associated with other immune-mediated diseases, such as autoimmune thyroiditis, T1DM, SLE, and jSSc, than in the general population [1,24,25,26], the exclusion of any diagnosed connective tissue disease in our cohort is particularly noteworthy. Although systemic autoantibodies are known to contribute to vascular damage in connective tissue disorders such as SLE [27], our findings did not reveal significant differences in capillaroscopic features between ANA-positive and ANA-negative children with CD. ANA testing is widely used as an adjunctive tool in the evaluation of autoimmune diseases, especially SLE and other rheumatic disorders, with a negative result providing strong evidence against active autoimmunity [28]. While increased ANA positivity has been reported in CD [29], its clinical significance in the absence of coexisting autoimmune diseases remains unclear. In CD, ANA positivity may result from a genetic predisposition to autoimmunity (e.g., HLA-DQ2/DQ8), chronic mucosal inflammation with subsequent antigen spreading and nuclear antigen exposure in the gut, coexisting autoimmune disorders, or nonspecific polyclonal B cell activation during chronic immune stimulation [30]. Indeed, ANA positivity on its own is neither pathognomonic nor diagnostic for a specific autoimmune condition, as it may be present in up to 15% of children in the general population [31] and should always be interpreted within the broader clinical context. Taken together, these results suggest that the microvascular changes observed in CD are unlikely to be driven by systemic autoantibodies such as ANA.

Endothelial cells are central components of inflammation, acting both as targets and mediators [32]. Previous studies have shown elevated serum levels of several proinflammatory cytokines, which correlate with IgA anti-TG2 titers and villous atrophy, as well as increased endothelial adhesion molecules in pediatric CD patients [22,33], potentially indicating microvascular damage. Furthermore, CD-related autoantibodies, particularly anti-tTG, may impair endothelial function and inhibit angiogenesis, leading to vascular disorganization and immaturity in the small intestinal mucosa of untreated patients [34]. A recent study aiming to clarify the mechanisms of autoantibody formation in CD demonstrated that IgA-switched TG2-specific B cells are uniquely present in untreated patients and are distinct from those observed in autoimmune diseases—most notably SLE—and in particular chronic infectious diseases [35]. These findings support a gluten-driven pathway for organ-specific autoantibody production in CD, distinct from the mechanisms underlying autoimmune connective tissue diseases such as SLE. Collectively, these findings suggest that microvascular involvement in CD may arise through disease-specific pathways, highlighting important directions for future research.

NVC is a validated, easy-to-use, non-invasive technique for microvascular assessment in rheumatologic diseases. It provides immediate visual results during outpatient evaluation [9,16,17]. In pediatric rheumatology, NVC is a key imaging modality for jSSc, with diagnostic value in evaluating Raynaud’s phenomenon as part of the jSSc criteria [16]. It has also proven useful in monitoring treatment response, particularly in JDM [9]. From a gastroenterology perspective, interest in NVC is limited but increasing in inflammatory bowel disease [11]. Our findings indicate that NVC could also be a valuable tool in CD, as it differentiated the capillary pattern from that of HCs. Further research is warranted to establish a cost-effective strategy for disease monitoring and for assessing adherence to GFD, potentially in combination with tTGA measurement. If confirmed in larger prospective studies, this approach could be integrated into routine follow-up to enable early detection of systemic changes, identify potential coexisting autoimmune diseases such as rheumatologic or endocrinologic disorders, and assess GFD adherence. This may help to optimize patient management and improve long-term outcomes in pediatric CD.

There were some limitations to this study, primarily the cross-sectional design, which prevented the assessment of the longitudinal progression or reversibility of NVC abnormalities following dietary intervention. A second limitation was that the relatively small number of participants may limit the extent to which these findings can be generalized to the broader pediatric CD population. Third, ethical constraints prevented the use of serological tests and intestinal biopsies in healthy control subjects, so the possibility of undetected subclinical CD in this group cannot be entirely ruled out. As this can be considered a pilot study, all NVC assessments were performed by a single evaluator, and inter- or intra-observer reliability could not be assessed. Future studies should incorporate independent evaluations by multiple observers to assess and improve measurement reliability. Additionally, although the presence of nonspecific NVC patterns was significantly more frequent in CD patients compared to healthy controls, their nonspecific nature limits the potential use of NVC as a diagnostic tool. Finally, the lack of long-term clinical and NVC follow-up restricts the ability to determine the prognostic value of capillaroscopic findings in predicting future autoimmune or vascular complications. Nevertheless, to the best of our knowledge, this is the first report to have evaluated NVC findings in pediatric CD patients. The findings obtained indicate the potential role of NVC as a non-invasive and cost-effective method for assessing microvascular involvement and for screening coexisting autoimmune conditions in children with CD.

## 5. Conclusions

This paper provides the first evidence of microvascular alterations in pediatric CD as assessed by NVC. The observed abnormalities, notably reduced capillary density and increased morphological changes, suggest potential microvascular involvement, possibly driven by disease-specific immune mechanisms. Associations between these microvascular changes and factors such as tTG-IgA levels, patient age, and disease duration indicate that both ongoing disease activity and long-term immune-mediated damage may contribute to their development. Although the study results indicate that NVC has potential as a practical, non-invasive approach in the detection of vascular alterations in children with celiac disease, the nonspecific abnormalities identified can also occur in various autoimmune and non-autoimmune conditions, and their clinical significance remains uncertain. There is a need for long-term follow-up studies to investigate whether the microvascular changes observed are reversible, the mechanisms driving them, and whether they can provide insight into the course or prognosis of the disease. In addition, future research using standardized semi-quantitative scoring systems could enhance the comparability of results across studies.

## Figures and Tables

**Table 1:** Characteristics of study participants.

Variables	Celiac Disease (*n*:76)	HCs (*n*:76)	*p* Value
Age years, median (min–max)	13 (4–18)	11 (5–17)	0.08 ^µ^
Gender (male/female)	32/44	39/37	0.25 *
BMI-SDS median (min–max)	0.2 (−3.2–2.1)	0.45 (−2.1–2.2)	0.005 ^µ^
Disease duration months, median (min–max)	23.5 (0–156)	-	-
tTG-IgA value U/mL, median (min–max)	52 (10–200)	-	-
Patients without tTG-IgA normalization, *n* (%)	44 (57.9)	-	-
ANA: Negative *n* (%)	66 (86.8)	-	-

BMI: body mass index; SDS: Standard Deviation Score; HCs: healthy controls; tTG-IgA: tissue transglutaminase IgA; ANA: antinuclear antibody. ^µ^ Mann Whitney U test. * Pearson Chi-Square test with Yates’ correction.

**Table 2:** Comparison of capillaroscopic findings between patients and controls.

Variables	Celiac Disease (*n*:76)	HCs (*n*:76)	*p* Value
Capillaroscopic Findings (per mm)			
Capillary density, median (IQR)	7(6.813–7.063)	7.063 (7–7.125)	<0.001 ^µ^
Dilated capillaries, median (IQR)	0 (0–0.094)	0 (0–0)	<0.001 ^µ^
Abnormal shapes, median (IQR)	0.13 (0–0.19)	0 (0–0)	<0.001 ^µ^
Crossing capillaries, median (min–max)	0.184 (0–0.938)	0 (0–0.375)	<0.001 ^µ^
Tortuous capillaries, median (min–max)	0.125 (0–0.93)	0 (0–0.313)	<0.001 ^µ^
Capillary length (µm), median (IQR)	275 (246.5–306.5)	255 (230–290)	0.026 ^µ^
Reduced capillary density (<7), *n* (%)	33 (43.4)	5 (6.6)	<0.001 *
Presence of dilated capillaries, *n* (%)	25 (32.9)	3 (3.9)	<0.001 *
Presence of giant capillaries, *n* (%)	0	0	>0.05
Presence of abnormal shapes, *n* (%)	45 (59.2)	0 (0)	<0.001 *
Presence of microhemorrhages, *n* (%)	8 (10.5)	0 (0)	0.006 *
Presence of crossing capillaries, *n* (%)	62 (81.6)	15 (19.7)	<0.001 *
Presence of tortuous capillaries, *n* (%)	45 (59.2)	19 (25)	<0.001 *
Overall NVC pattern			
Normal pattern, *n* (%)	22 (28.9)	68 (89.5)	<0.001 *
Nonspecific pattern, *n* (%)	54 (71.1)	8 (10.5)	

NVC: Nailfold videocapillaroscopy, HCs: healthy controls, IQR: interquartile range. ^µ^ Mann–Whitney U test. * Pearson Chi-Square test, Yates’ correction, Fisher’s exact test.

**Table 3:** Comparison of capillaroscopic parameters among patient subgroups and healthy controls.

Capillaroscopic Parameters (per mm)	Group 1 (*n*:44)	Group 2 (*n*:32)	HCs (*n*:76)	*p* ^Ƙ^ Value	*p* ^1−2^	*p* ^1−3^	*p* ^2−3^
Capillary length (µm), median (IQR)	279.5 (242.5–325.5)	270 (249–302)	255 (230–290)	0.081	-	-	-
Capillary density, median (IQR)	6.93 (6.81–7.06)	7 (6.87–7)	7.06 (7–7.12)	<0.001	>0.999	<0.001	0.003
Dilated capillaries, median (IQR)	0 (0–0.12)	0 (0–0.03)	0 (0–0)	<0.001	0.33	<0.001	0.029
Capillaries with abnormal shape, median (IQR)	0.12 (0–0.18)	0 (0–0.18)	0 (0–0)	<0.001	0.022	<0.001	<0.001
Crossing capillaries, median (min–max)	0.18 (0–0.93)	0.12 (0–0.68)	0 (0–0.37)	<0.001	>0.999	<0.001	<0.001
Tortuous capillaries, median (min–max)	0.12 (0–0.93)	0.12 (0–0.62)	0 (0–0.03)	<0.001	>0.999	<0.001	0.001

Group 1 included patients without tTG-IgA normalization (>10 U/mL) and with symptoms suggestive of gluten exposure. Group 2 included those with normalized tTG-IgA (≤10 U/mL) and no symptoms. HCs: healthy controls, IQR: interquartile range. ^Ƙ^ Kruskal–Wallis H test, Dunn test, post hoc Bonferroni correction. The numbers used in the representation of post hoc analysis results are as follows: *p*^1−2^: Comparison of Group 1 and Group 2; *p*^1−3^: Comparison of Group 1 and HCs; *p*^2−3^: Comparison of Group 2 and HCs.

## Data Availability

All data generated or analyzed during this study are included in this published article. Additional information can be obtained from the corresponding author upon reasonable request. The data are not publicly available due to patient privacy concerns and institutional data protection policies.
